# Latent profile analysis of sleep quality and associated factors on sleep patterns in night-shift nurses: a cross-sectional study

**DOI:** 10.3389/fpubh.2026.1800662

**Published:** 2026-05-20

**Authors:** Xin Chu, Juan Lv, Guiling Li, Dawei Wang

**Affiliations:** 1 Department of Nephrology, The Affiliated Hospital of Qingdao University, Qingdao, China; 2 Department of Obstetrics and Gynecology, Qingdao Central Hospital, University of Health and Rehabilitation Sciences, Qingdao, China; 3 Department of Obstetrics, The Affiliated Hospital of Qingdao University, Qingdao, China

**Keywords:** latent profile analysis, nurse, shift work, sleep pattern, sleep quality

## Abstract

**Objective:**

This study aimed to investigate the potential categories of sleep quality in shift nurses using latent profile analysis, and to explore the characteristics and associated factors of sleep quality in each category.

**Methods:**

Convenience sampling was used to select 1,650 shift nurses from four tertiary A-level hospitals from May to June 2025. Data were collected using the Pittsburgh Sleep Quality Index (PSQI), the sleep chronotype questionnaire, the perceived stress scale, and a general demographic questionnaire. Latent profile analysis was conducted to determine the latent categories of sleep quality. Univariate and multinomial logistic regression analyses were used to investigate the associated factors of different sleep quality categories. A total of 1,650 questionnaires were distributed, of which 1,608 were valid, yielding a valid response rate of 97.5%.

**Results:**

The mean PSQI score among shift nurses was 7.23 ± 3.16 points, indicating overall sleep problems within this group. The sleep quality of night-shift nurses was categorized into four latent profiles: “good sleep quality” (44.4%), “moderate sleep quality” (40.7%), “sleep disorder–low sleeping pills” (9.6%), and “sleep disorder–high sleeping pills” (5.3%). A multivariate logistic regression analysis revealed that several factors were significantly associated with these sleep quality profiles. These factors included perceived stress, sleep chronotype, monthly night shifts, weekly working hours, age, and years of nursing experience, and marital status (*p* < 0.05).

**Conclusion:**

The sleep quality of shift-working nurses varies significantly. Nursing administrators should develop tailored management strategies and adopt targeted sleep interventions based on the identified patterns in nurses’ sleep quality to improve their overall sleep quality.

## 1 Introduction

In healthcare, the shift work system plays a crucial role in ensuring the continuity and safety of patient care during both day and night. However, this shift work schedule and the highly stressful working environment often disrupt nurses’ circadian rhythms, making it challenging for night-shift nurses to get enough rest and high-quality sleep (1–3). Poor sleep quality among shift nurses has become a widespread and serious occupational health issue. Sleep quality is associated with multiple factors, including individual differences in circadian rhythms, work-related stress, and working hours (4–7).

Shift nurses experience significantly higher rates of sleep disorders, characterized by difficulty falling asleep, interrupted sleep, and poor overall sleep quality (8). These issues mainly arise from irregular work schedules and high stress levels, including long night shifts and frequent shift rotation. Night shifts disrupt normal circadian rhythms, making it difficult for nurses to get adequate rest during the day or night, ultimately affecting the quality and depth of their sleep (9–11). Sleep disorders not only impair nurses’ quality of life but also significantly compromise their job performance. Studies show that insufficient sleep and fatigue reduce nurses’ work efficiency, increase the risk of medical errors, and adversely impact patient safety and treatment outcomes (12, 13). Therefore, it is crucial to assess the sleep quality of shift nurses, implement interventions to improve it, and ultimately enhance their work efficiency, quality of life, and the overall quality of nursing care.

Among tools for assessing sleep quality, the Pittsburgh Sleep Quality Index (PSQI) is widely recognized for its comprehensiveness, objectivity, and reliability (14). However, many existing studies rely heavily on the PSQI as the sole criterion for evaluating sleep quality, often overlooking the differences among individuals. The majority of studies focus on a general analysis of sleep quality and lack targeted strategies to prevent or intervene in sleep disorders. Latent profile analysis (LPA) is a person-centered statistical approach that identifies heterogeneous latent subgroups based on multiple continuous indicators. Its main advantages include capturing unobserved population heterogeneity, avoiding arbitrary classification thresholds, reflecting holistic response patterns, and providing model-based statistical evidence, thereby offering a more accurate understanding of individual differences than traditional variable-centered methods. The PSQI total score provides only a global index of sleep quality and fails to capture heterogeneous patterns of sleep disturbance across components. Individuals with the same total scores may differ substantially in terms of sleep latency, duration, and efficiency. A person-centered approach (LPA) identifies subgroups based on multidimensional response patterns rather than total scores, revealing clinically meaningful subtypes that are obscured by variable-centered total-score approaches. Thus, LPA is superior to reliance on the PSQI total score alone (15–17). A deeper understanding of the latent characteristics of different sleep quality profiles in shift nurses can help in recognizing individual differences, ultimately developing a more targeted management strategy.

This study aims to apply latent profile analysis to identify the potential categories of sleep quality among shift nurses. It will also analyze the characteristics and factors associated with sleep quality in each category. It is expected to provide more scientific and targeted guidance for nursing managers, allowing them to effectively support shift nurses and improve their sleep quality.

## 2 Materials and methods

### 2.1 Design

This was an exploratory, quantitative, person-centered, cross-sectional study. Convenience sampling was used to recruit participants.

### 2.2 Participants

Night-shift nurses were recruited from four branch hospitals of the affiliated Hospital of Qingdao University (tertiary A-level) between May and June 2025. The inclusion criteria were as follows: (1) registered nurses, (2) having at least 1 year of night shift work experience, (3) currently working night shifts, and (4) providing voluntary participation in the survey with written informed consent. The exclusion criterion were as follows: (1) being on leave during the survey period; (2) being absent from work due to illness, maternity leave, or external training; (3) nurses with long-term sleep medication use who have been using sleep aids for an extended period prior to their work (daily or near-daily use for more than 3 months); and (4) head nurses or nurses working exclusively daytime shifts. This study was approved by the Ethics Committee of the Affiliated Hospital of Qingdao University. All participants provided written informed consent.

### 2.3 Research instruments

#### 2.3.1 General information questionnaire

**Figure 1:**
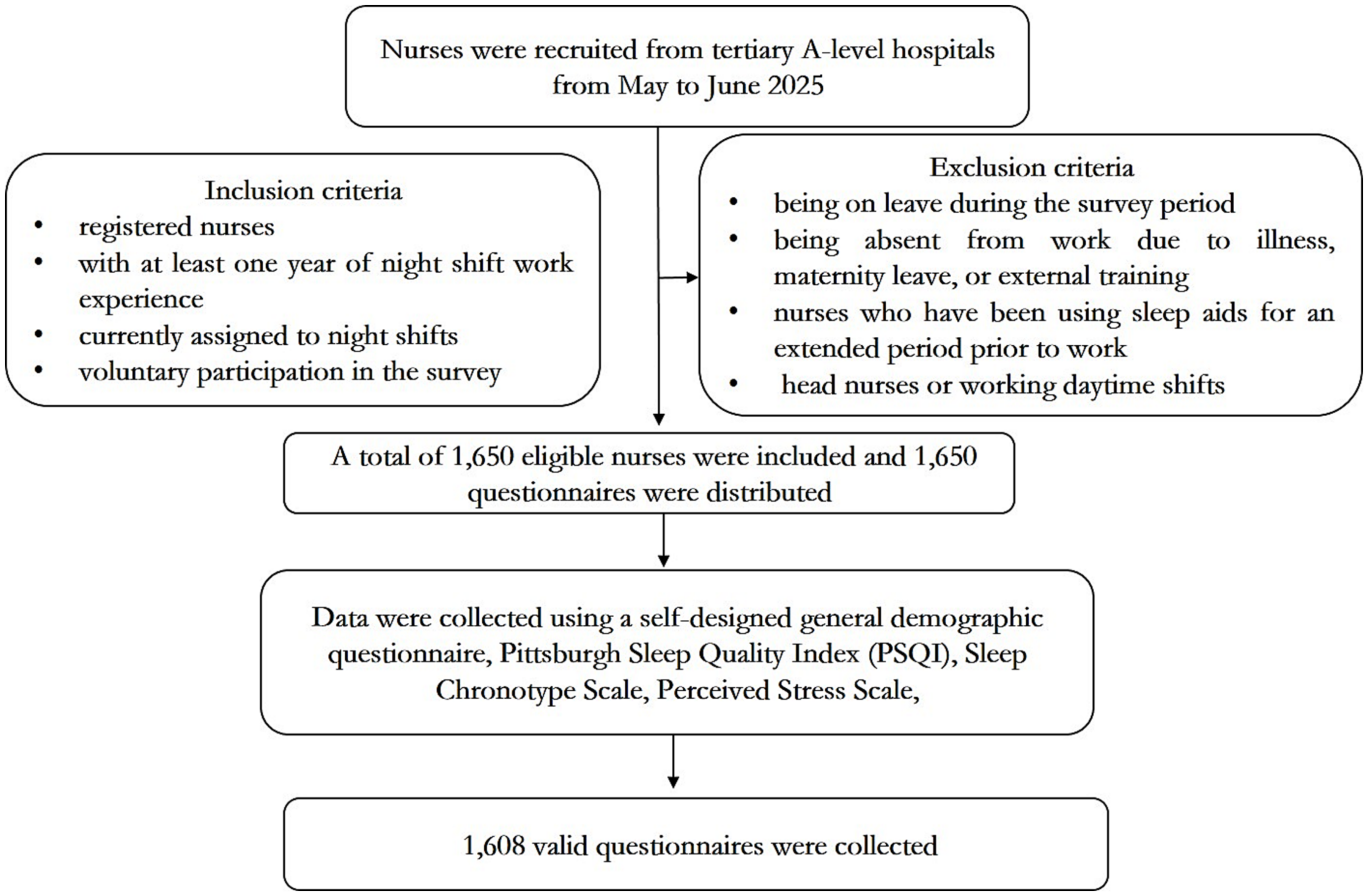
A flowchart of participants.

A self-designed demographic questionnaire was developed to collect general information from shift nurses. This included sociodemographic characteristics (age, sex, educational level, marital status, and number of children), and shift work-related attributes (affiliated department, professional title, years of nursing experience, monthly night shifts, and weekly working hours) (18). A flowchart of participant recruitment is illustrated in Figure 1.

#### 2.3.2 PSQI

The sleep quality of shift nurses was assessed using the PSQI, originally developed by Buysse in 1989 (19). This scale consists of seven dimensions: subjective sleep quality, sleep latency, sleep duration, sleep efficiency, sleep disturbances, use of sleep medication, and daytime dysfunction. It is typically used to evaluate the participants’ sleep status over the past month. Each dimension is scored from 0 to 3, yielding a total PSQI score ranging from 0 to 21. A total score greater than 7 (cutoff value = 7) indicates the presence of a sleep disorder, with higher scores indicating poorer sleep quality. The Chinese version of the PSQI demonstrates good reliability, with a Cronbach’s alpha coefficient of 0.72 (20).

#### 2.3.3 Perceived stress scale (PSS)

Perceived Stress Scale was utilized to assess the perceived stress of the shift nurses (21, 22). The original 14-item scale (PSS-14), developed by Cohen in 1983, consists of 7 positive items and 7 negative items, each rated on a 5-point Likert scale. The total score ranges from 0 to 56, with higher scores indicating a higher level of perceived stress. A total score greater than 25 suggests that the individual is experiencing stress that is potentially harmful to health. The Cronbach’s *α* coefficient for the Chinese version of this scale is 0.88 (23).

#### 2.3.4 Sleep chronotype scale

This study used the simplified sleep chronotype scale developed by Adan and Almirall to evaluate individual differences in sleep chronotype (24). This scale consists of 5 questions, yielding a total score ranging from 4 to 25. A higher score indicates a stronger inclination toward being an early morning type. Specifically, the scoring categories are as follows: 4–7 points indicate an absolute evening type; 8–11 points, a moderate evening type; 12–17 points, an intermediate type; 18–21 points, a moderate morning type; and 22–25 points, an absolute morning type. The Cronbach’s *α* coefficient for this scale in this study was 0.90.

### 2.4 Data collection

The online questionnaire survey was conducted using Questionnaire Star, and responses were collected through WeChat. Before participants began answering the questions, the researcher explained the purpose and content of the study to the shift nurses. Only those who met the requirements were invited to participate. To prevent duplicate submissions, each account and device was limited to one response. After completing the survey, two researchers individually verified the responses and removed any questionnaires that did not meet the criteria.

### 2.5 Statistical analysis

Latent profile analysis (LPA) was conducted using Mplus 8.0 software. The model fit was assessed using several fit indices. Lower values of Akaike Information Criterion (AIC), Bayesian Information Criterion (BIC), and adjusted BIC (aBIC) indicate a better model fit. The Lo–Mendell–Rubin adjusted likelihood ratio test (LMRT) and bootstrap likelihood ratio test (BLRT) were used to compare the fit differences between the k-1 model and k model. A *p*-value of less than 0.05 indicates that the k-profile model is superior to the k-1 profile model. Entropy was used to evaluate classification accuracy, with values closer to 1 indicating higher classification accuracy (25).

Statistical analysis was performed using SPSS 25.0 software. For measurement data that conformed to a normal distribution, results were expressed as mean ± standard deviation (*x* ± *s*), and comparisons among groups were performed using one-way analysis of variance. The count data were expressed as frequencies and percentages, and comparisons among groups were performed using the chi-squared test. A multivariable logistic regression analysis was conducted to examine factors associated with sleep quality in different categories. A *p*-value of less than 0.05 was considered statistically significant.

The proportion of missing data in this study was minimal (all <5%). Therefore, listwise deletion was applied to handle missing data without introducing significant bias. Assumptions of the multinomial logistic regression model were assessed. Multicollinearity among independent variables was evaluated using the variance inflation factor (VIF). A VIF value of less than 10 was considered acceptable, indicating no evidence of severe multicollinearity.

## 3 Results

### 3.1 General information about shift nurses

A total of 1,650 questionnaires were distributed, of which 1,608 were valid, yielding a valid response rate of 97.5%. Among them, 56 were male nurses (3.5%), and 1,552 were female nurses (96.5%). The average age of the nurses was 33.65 ± 4.58 years old. For marital status, 896 nurses (55.7%) were married, while 712 (44.3%) were unmarried. Regarding nursing experience, 957 nurses (59.5%) had less than 10 years of nursing experience, while 651 (40.5%) had more than 10 years. Regarding the frequency of monthly night shifts, 170 (10.6%) had no more than 5 night shifts, 720 (44.8%) had 6-to-10-night shifts, and 718 (44.6%) had more than 10 night shifts (34.50%). The demographic information is shown in Table 1.

**Table 1:** Demographic information of night-shift nurses (*n* = 1,608).

Predictor	Total (*n* = 1,608)	Good sleep [*n* (%)] (714) 44.4%	Moderate sleep [*n* (%)] (654) 40.7%	Sleep disorder -low sleeping pills [*n* (%)] (154) 9.6%	Sleep disorder -high sleeping pills [*n* (%)](86)5.3%	*χ* ^2^	*p*
Age (years)						17.572	0.007
20–30	628 (39.1)	300 (42.0)	249 (38.1)	55 (35.7)	24 (27.9)		
31–40	896 (55.7)	378 (52.9)	379 (58.0)	87 (56.5)	52 (60.5)		
>40	84 (5.2)	36 (5.1)	26 (3.9)	12 (7.8)	10 (11.6)		
Sex						4.505	0.212
Male	56 (3.5%)	32 (4.5%)	19 (2.9%)	4 (2.6)	1 (1.1)		
Female	1,552 (96.5%)	682 (95.5%)	635 (97.1)	150 (97.4)	85 (98.9)		
Education level						2.339	0.505
College or below	265 (16.5)	107 (15.0)	118 (18.0)	26 (16.9)	14 (16.3)		
Bachelor’s degree or above	1,343 (83.5)	607 (85.0)	536 (82.0)	128 (83.1)	72 (83.7)		
Marital status						31.769	<0.001
Unmarried	712 (44.3)	350 (49.0)	294 (45.0)	46 (30.0)	22 (25.6)		
Married	896 (55.7)	364 (51.0)	360 (55.0)	108 (70.0)	64 (74.4)		
Department affiliation						17.639	0.007
Emergency	234 (14.6)	86 (12.0)	98 (15.0)	31 (20.1)	19 (22.1)		
ICU	250 (15.5)	100 (14.0)	105 (16.0)	28 (18.2)	17 (19.8)		
others	1,124 (69.9)	528 (74.0)	451 (69.0)	95 (61.7)	50 (58.1)		
Professional title						14.412	0.025
Junior nurse	168 (10.4)	93 (13.0)	59 (9.0)	12 (7.8)	4 (4.7)		
Senior nurse	673 (41.9)	300 (42.0)	281 (43.0)	58 (37.7)	34 (39.5)		
Supervising nurse or above	767 (47.7)	321 (45.0)	314 (48.0)	84 (54.5)	48 (55.8)		
Years of nursing experience						17.466	<0.001
≤10	957 (59.5)	450 (63.0)	392 (59.9)	76 (49.4)	39 (45.3)		
>10	651 (40.5)	264 (37.0)	262 (40.1)	78 (50.6)	47 (54.7)		
Weekly working hours						12.874	0.005
≤40	208 (12.9)	108 (15.1)	85 (13.0)	11 (7.1)	4 (4.7)		
>40	1,400 (87.1)	606 (84.9)	569 (87.0)	143 (92.9)	82 (95.3)		
Monthly night shifts						35.505	<0.001
≤5	170 (10.6)	98 (13.7)	65 (10.0)	11 (7.1)	3 (3.5)		
6–10	720 (44.8)	336 (47.1)	294 (45.0)	59 (38.3)	26 (30.2)		
>10	718 (44.6)	280 (39.2)	295 (45.0)	84 (54.6)	57 (66.3)		
Sleep chronotype						23.224	<0.001
Morning type	397 (24.7)	207 (29)	150 (23)	27 (17.5)	13 (15.1)		
Intermediate type	497 (30.9)	221 (31)	209 (32)	43 (28)	24 (27.9)		
Evening type	714 (44.4)	286 (40)	295 (45)	84 (54.5)	49 (57.0)		
Score of perceived stress	38.98 ± 9.76	36.56 ± 7.26	40.08 ± 9.62	42.19 ± 10.28	43.36 ± 10.56	45.125	<0.001

### 3.2 Latent profile analysis of sleep quality in shift nurses

A total of five different latent profile models were fitted based on the scores from the seven components of the PSQI scale. The model with lower AIC, BIC, and aBIC values would indicate a better fit, whereas the model with an entropy value approaching 1 would indicate a clear delineation of the classes. A significant LMRT or BLRT (with *p* < 0.05) indicated that the k-class model outperformed the k-1 class model in terms of model fit. We relied on a combination of statistical indices and substantive interpretation in comparing competing models with different numbers of classes and used LMRT and BLRT as significance tests to compare models with different profiles. The model fit indices are shown in Table 2. Across models 1 to 5, the AIC, BIC, and aBIC values decreased as the number of profiles increased, with entropy values exceeding 0.900. Although the 5-class model exhibited slightly lower AIC and BIC values, it yielded one extremely small class comprising less than 5% of the total sample, and the LMR *p*-value was greater than 0.05. In contrast, each class in the 4-class model was sufficiently sized. The values of AIC, BIC, and aBIC were close to their minimum; the *p*-values for LMR and BLRT were less than 0.05; and it exhibited the highest entropy value. Therefore, the 4-profile model was identified as the optimal model.

**Table 2:** Fit indicators of sleep quality in the latent profile model.

Model	AIC	BIC	aBIC	Entropy	LMR(P)	BLRT(P)	Probability
1	21256.12	21332.45	21306.78	–	–	–	1.00
2	16209.54	16325.76	16252.05	0.954	0.2356	<0.001	0.910/0.090
3	14820.16	14972.46	14862.34	0.992	0.0045	<0.001	0.053/0.857/0.096
4	13675.45	13856.98	13736.28	0.998	0.0060	<0.001	0.444/0.407/0.096/0.053
5	13057.20	13295.32	13165.38	0.962	0.2500	<0.001	0.048/0.425/0.442/0.053/0.032

### 3.3 Naming the latent profiles of sleep quality

**Figure 2:**
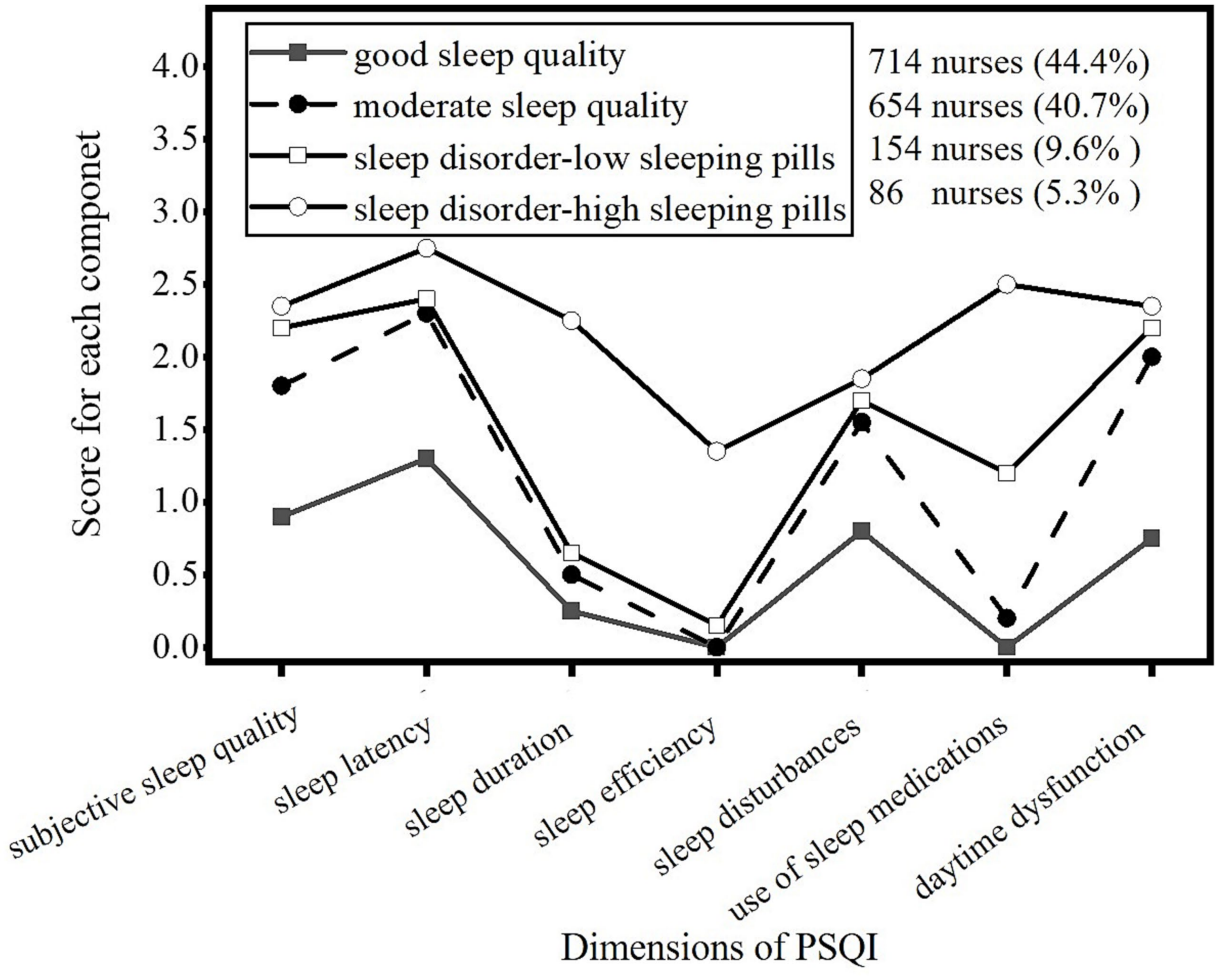
Distribution for each dimension of PSQI in the four latent categories.

Latent profiles of sleep quality were named based on Model 4. Shift nurses’ sleep quality can be categorized into four distinct classes: good sleep quality (C1), moderate sleep quality (C2), sleep disorder with low use of sleeping pills (C3), and sleep disorder with high use of sleeping pills (C4). Figure 2 illustrates the distribution of PSQI across each dimension in the four latent categories. C1 consists of 714 nurses, representing 44.4% of the total sample. This class exhibited relatively low scores across all dimensions, indicating good sleep quality, and was therefore labeled “good sleep quality.” C2 includes 654 nurses, accounting for 40.7% of the sample. This group showed medium-to-high scores on all dimensions of the PSQI scale, reflecting moderate overall sleep quality, and was thus named “moderate sleep quality.” C3 includes 154 nurses (9.6%) and was labeled “sleep disorder–low sleeping-pill use.” C4 consists of 86 nurses, representing 5.3% of participants. This class showed high scores in sleep quality, sleep medication use, and daytime dysfunction, indicating significant sleep disorders. Consequently, it was named “sleep disorder–high sleeping-pill use.”

### 3.4 Univariate analysis of factors associated with sleep quality

The average PSQI score for 1,608 shift nurses was 7.23 ± 3.16 points. Among them, the PSQI scores for nurses with good sleep, moderate sleep, sleep disorder–low sleeping pills, and sleep disorder–high sleeping pills were 5.16 ± 1.55, 6.80 ± 1.50, 13.10 ± 2.25, and 15.40 ± 2.65 points, respectively. The univariate analysis revealed that several factors were associated with sleep quality in different latent profiles (*p* < 0.05), including age, professional title, years of working experience, weekly working hours, monthly night shift, and perceived stress score.

### 3.5 Multinomial logistic regression analysis of factors associated with sleep quality

The four latent profiles of sleep quality were treated as the dependent variable, and the variables identified as statistically significant in the univariate analysis were defined as independent variables. The good-sleep type was set as the reference group, and a multinomial logistic regression analysis was performed. A parallel test was performed to examine the proportional odds assumption. The result showed *p* = 0.008, which is < 0.05, indicating a significant violation of the proportional odds assumption. Therefore, ordered logistic regression was not appropriate, and multinomial logistic regression was used instead, as it does not require the proportional odds assumption. The assignment methods for the independent variables are presented in Table 3. The model likelihood ratio χ2 = 305.36 (*p* < 0.01) indicated that perceived stress, monthly night shifts, weekly working hours, and years of nursing experience were factors associated with the sleep quality of shift nurses (*p* < 0.05), as shown in Table 4.

**Table 3:** Assignment of independent variables for the multinomial logistic regression analysis.

Variables	Assignment
Perceived stress	Original values
Sleep chronotype	Morning type = 1,intermediate type = 2, evening type = 3
Age (years)	20–30 years = 1, 31–40 years = 2, >40 years = 3
Marital status	Married = 1, unmarried = 2
Years of nursing experience	≤10 = 1, >10 = 2
Monthly night shifts	≤5 = 1, 6–10 = 2, >10 = 3
Weekly working hours	≤40 = 1, >40 = 2
Department affiliation	Emergency = 1, ICU = 2, others = 3
Professional Title	Junior nurse = 1, senior nurse = 2, supervising nurse or above = 3

**Table 4:** Multinomial logistic regression analysis of factors associated with sleep quality profiles.

Variables	β	SE	Wald *χ*^2^	*p*	OR	95%CI
C2 vs. C1 (C1 as reference)					–	–
	−3.731	0.506	54.400	<0.001		
Perceived stress	0.768	0.304	6.402	0.011	2.156	(1.189–3.909)
Monthly night shifts (ref: <5)						
6–10	1.049	0.371	7.977	0.005	2.854	(1.378–5.910)
>10	1.162	0.341	11.650	0.001	3.197	(1.640–6.232)
Sleep chronotype (ref: morning type)						
Intermediate type	0.981	0.332	8.731	0.003	2.666	(1.391–5.110)
Evening type	0.849	0.367	5.339	0.021	2.337	(1.138–4.800)
Age (ref: 20–30 years old)						
31–40 years old	1.039	0.357	8.483	0.004	2.826	(1.405–5.685)
>40 years old	1.176	0.395	8.864	0.003	3.241	(1.495–7.029)
Marital status (ref: unmarried)						
Married	1.040	0.327	10.128	0.001	2.829	(1.491–5.367)
Years of nursing service (ref: <10 years)						
>10 years	0.913	0.302	9.119	0.003	2.492	(1.378–4.508)
Weekly working hours (ref:≤40 h)						
>40 h	0.638	0.311	4.217	0.040	1.893	(1.029–3.480)
C3 vs. C1 (C1 as reference)						
	−3.945	0.539	53.569	<0.001		
Perceived stress	0.882	0.318	7.718	0.005	2.417	(1.297–4.504)
Monthly night shifts (ref: <5)						
6–10	0.905	0.363	6.225	0.013	2.473	(1.214–5.036)
>10	0.959	0.383	6.267	0.012	2.609	(1.231–5.526)
Sleep chronotype (ref: morning type)						
Intermediate type	0.941	0.357	6.942	0.008	2.563	(1.273–5.163)
Evening type	0.964	0.383	6.328	0.012	2.621	(1.237–5.553)
Age (ref: 20–30 years old)						
31–40 years old	0.862	0.375	5.278	0.022	2.369	(1.135–4.944)
>40 years old	1.087	0.412	6.952	0.008	2.966	(1.322–6.654)
Marital status (ref: unmarried)						
Married	1.238	0.340	13.225	<0.001	3.449	(1.770–6.722)
Years of nursing service (ref: <10 years)						
>10 years	1.028	0.320	10.328	0.001	2.795	(1.493–5.232)
Weekly working hours (ref:≤40 h)						
>40 h	0.670	0.325	4.251	0.039	1.955	(1.034–3.698)
C4 vs. C1 (C1 as reference)						
	−5.240	0.724	52.307	<0.001		
Perceived stress	1.043	0.399	6.812	0.009	2.837	(1.297–6.206)
Monthly night shifts (ref: <5)						
6–10	1.248	0.482	6.708	0.010	3.483	(1.355–8.956)
>10	1.340	0.495	7.310	0.007	3.818	(1.446–10.082)
Sleep chronotype (ref: morning type)						
Intermediate type	1.070	0.473	5.120	0.024	2.916	(1.154–7.366)
Evening type	1.137	0.490	5.383	0.020	3.117	(1.193–8.145)
Age (ref: 20–30 years old)						
31–40 years old	0.936	0.466	4.027	0.045	2.549	(1.022–6.358)
>40 years old	1.066	0.523	4.152	0.042	2.903	(1.041–8.090)
Marital status (ref: unmarried)						
Married	1.145	0.429	7.128	0.008	3.142	(1.356–7.279)
Years of nursing service (ref: <10 years)						
>10 years	0.936	0.409	5.251	0.022	2.550	(1.145–5.680)
Weekly working hours (ref:≤40 h)						
>40 h	1.091	0.409	7.119	0.008	2.976	(1.336–6.630)

C1, good sleep quality; C2, moderate sleep quality; C3, sleep disorder–low sleeping-pills use; C4, sleep disorder–high sleeping-pills use; OR, odds ratio; CI, confidence interval.

## 4 Discussion

In this study, the PSQI score for 1,608 shift nurses was 7.23 ± 3.16 points, indicating that shift nurses generally experience sleep problems, consistent with the results of other studies (17). Shift nurses often have demanding schedules during the day, frequently work night shifts, and carry significant responsibilities, all of which can disrupt their circadian rhythms and lead to sleep disorders (26).

Using LPA, this study identified four latent profiles of sleep quality in shift nurses, namely good sleep quality (C1), moderate sleep quality (C2), sleep disorder–low sleeping-pill use (C3), and sleep disorder–high sleeping-pill use (C4). The results revealed differences in PSQI scores among these categories, effectively reflecting the varying sleep statuses of shift nurses. Among the four categories, C1 accounted for 44.4% of shift nurses. This category was primarily characterized by younger nurses, those with less than 10 years of work experience, 6–10 night shifts per month, an intermediate sleep chronotype, and relatively low perceived stress levels. C2 comprised 40.7% of shift nurses, indicating that this group could flexibly adjust their sleep patterns to compensate for the sleep quality after adapting to a shift-work schedule. C3 accounted for 9.6% of shift nurses. Typical characteristics of this group included difficulty falling asleep, insufficient sleep, and low sleep efficiency. C4 accounted for 5.3% of shift nurses. Nurses in this category exhibited significantly poor sleep quality, reliance on hypnotic medications, and daytime functional disorders. Groups C3 and C4 were primarily characterized by high perceived stress, an evening chronotype, frequent night shifts (more than 10 shifts per month), over 10 years of nursing experience, and working more than 40 h per week.

### 4.1 Perceived stress

Clinical nursing tasks were heavy and complex, accompanied by significant work pressure and a high-stress work environment, which would hinder shift nurses from achieving good-quality sleep. This study found that perceived stress scores across the four categories of shift nurses were all above 25 points, indicating high levels of health-related stress. Those experiencing higher levels of perceived stress were more likely to encounter difficulty falling asleep, low sleep efficiency, or sleep disorders requiring medication, suggesting that they have been in a high-stress state for an extended period. This finding was consistent with that of Yao, who reported a negative correlation between higher stress levels and poorer sleep quality (9, 27, 28).

### 4.2 Sleep chronotype

Sleep chronotype was an important factor associated with sleep patterns. Individuals classified as absolute morning type were more likely to experience good sleep quality, while those identified as absolute night type tended to experience sleep disorders, with difficulties falling asleep and maintaining efficiency. Individuals with different circadian rhythms exhibited varying degrees of adaptability to shift work. This difference may be attributed to the fact that individuals with an absolute morning chronotype tend to go to bed and wake up earlier, whereas those with an absolute evening chronotype exhibit night-owl-like sleep patterns and often fail to achieve sufficient sleep recovery, thereby leading to poor sleep quality and decreased work efficiency. This finding was similar to that of Hakimi & Qu, who reported that an evening chronotype is associated with burnout and poor sleep quality (29, 30).

### 4.3 Age

This study indicated that older age was associated with poor sleep quality among shift nurses. Nurses over 40 years of age were more likely to be classified into the sleep disorder group, which was consistent with previous results (31, 32). With advancing age, it becomes more challenging for rotating night-shift nurses to adjust their circadian rhythms to variable sleep schedules. Reilly et al. (33) suggested that older individuals tend to shift to a morning chronotype rather than a night owl chronotype with aging. Moreover, most female nurses over 40 years of age are experiencing menopause, which can lead to fluctuations in hormone levels that adversely affect sleep quality.

### 4.4 Marital status

This study found that married night-shift nurses were more likely to be categorized into the “sleep disorder” group. Many married nurses devote substantial energy to heavy household chores and childcare. Their daytime sleep is frequently disturbed by family responsibilities and social activities. It is common for married nurses to bear greater responsibility and pressure at home, contributing to their poor sleep quality. The findings indicated that married nurses exhibited poorer sleep quality, which was inconsistent with some previous studies. Pan et al. (14) indicated that unmarried nurses were more likely to have poor sleep quality because of feelings of loneliness.

### 4.5 Years of nursing service

Years of nursing service were associated with sleep disorders among nurses. Those with fewer years of work experience were more likely to be classified in the good sleep quality group. This may be explained by their shorter professional experience and, consequently, lower levels of job- and family-related stress. With increasing years yeas of service, nurses’ sleep quality gradually deteriorated, making it difficult for them to achieve sufficient restorative sleep (34).

### 4.6 Monthly night shifts

Nurses with a lower frequency of night shifts were more likely to report good sleep quality. Nurses with more than 10 night shifts per month were more likely to belong to the “sleep disorder” group. This further confirmed that the sleep quality of shift nurses was negatively affected by frequent night shifts. This finding was consistent with previous studies (1, 4).

### 4.7 Weekly working hours

Nurses working more than 40 h per week were more likely to be classified into the sleep disorder group. This finding was consistent with previous studies reporting a correlation between longer working hours and poorer sleep quality among shift nurses (35, 36). A primary reason for this is the demanding nature of nursing tasks, which require long hours of intense concentration and physical endurance. Both physical and mental fatigue can increase the risk of poor sleep quality.

Given that shift work has been identified as a major factor associated with sleep disorders among shift nurses, it is essential to accurately identify sleep problems, develop targeted management strategies, and implement effective interventions. These measures will help improve shift nurses’ sleep patterns and quality, thereby promoting overall health and wellbeing.

### 4.8 Implications

From a policy perspective, our findings highlight the importance of flexible working conditions and strong support systems for nurses. Policies should prioritize developing a supportive and inclusive workplace environment by integrating flexible work schedules and stress management programs. This study provides both theoretical guidance and empirical evidence for the development of targeted interventions to enhance sleep quality. The findings highlight the necessity for targeted interventions in nursing practice and policy, based on the latent profiles and the demographic characteristics of nurses. Nurses from different profiles face distinct needs and challenges that require customized strategies. These targeted interventions could enhance nurses’ sleep quality, ultimately improving patient care and the sustainability of the healthcare system.

### 4.9 Limitations

This study has several limitations. First, it used a cross-sectional design, which did not allow for tracking the sleep quality of shift nurses over time. Second, participants were recruited through an online questionnaire platform, leaving it uncertain whether there were differences between those who participated and those who declined. Self-report bias may exist because all data, including sleep quality and related influencing factors, were collected through self-reported questionnaires rather than objective measurements. Finally, the findings have limited generalizability, as the sample was recruited exclusively from a single regional and single-institution setting. Future studies with larger sample sizes, multicenter designs, and objective sleep measurements are warranted to overcome these limitations.

## 5 Conclusion

In conclusion, this study used LPA to categorize the sleep quality of shift nurses into four subtypes: good sleep quality, moderate sleep quality, sleep disorder with low use of sleeping pills, and sleep disorder with high use of sleeping pills. The research revealed that sleep quality among shift nurses was associated with several factors, including age, marital status, monthly night shifts, weekly working hours, years of nursing experience, and perceived stress levels. This finding highlights the heterogeneity and diverse patterns of sleep quality among night-shift nurses. Nursing managers should develop tailored management strategies and implement targeted sleep interventions based on nurses’ sleep quality patterns to improve their sleep quality. Meanwhile, individual nurses should also adopt healthy behaviors and lifestyles to enhance their sleep quality.

## Data Availability

The original contributions presented in the study are included in the article/supplementary material, further inquiries can be directed to the corresponding author.
